# Supplementary material for: A Global Screen for Assembly State Changes of the Mitotic Proteome by SEC-SWATH-MS
Source: Cell Syst. 2020 Feb 26;10(2):133–155.e6. doi: 10.1016/j.cels.2020.01.001 (PMC7042714; doi:10.1016/j.cels.2020.01.001)

Q7Z4H3 | HDDC2_HUMAN | HDDC2 C6orf74 NS5ATP2 CGI−130
Monomer MW [kDa]: 23.39 Monomer expected elution fraction: 53

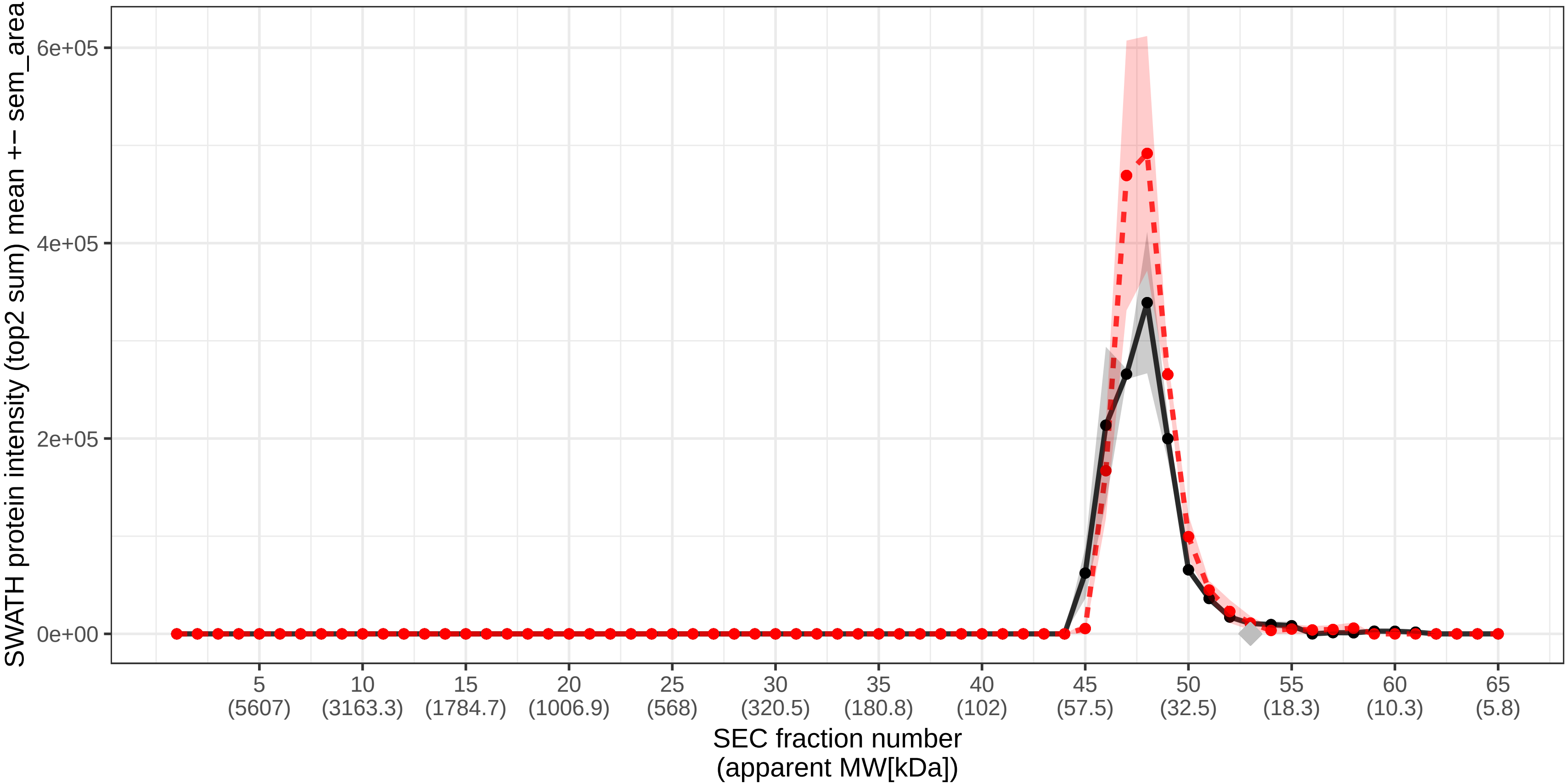

Supplement: Data S1. SEC-SWATH-MS Protein Chromatograms, Related to Figure 1 [file mmc6.zip › SECchrom_Q7Z4H3_HDDC2_HUMAN_HDDC2_C6orf74_NS5ATP2_CGI-130.pdf]
